# An Unusual Presentation of Multiple Superficial Mucoceles Occurring with Oral Lichen Planus

**DOI:** 10.1155/2021/2143829

**Published:** 2021-09-03

**Authors:** Dulyapong Rungraungrayabkul, Puangwan Lapthanasupkul, Naruemon Panpradit, Nis Okuma

**Affiliations:** ^1^Department of Oral Medicine and Periodontology, Faculty of Dentistry, Mahidol University, 6 Yothi Rd. Rajthewee, Bangkok, Thailand 10400; ^2^Department of Oral and Maxillofacial Pathology, Faculty of Dentistry, Mahidol University, 6 Yothi Rd. Rajthewee, Bangkok, Thailand 10400

## Abstract

Superficial mucoceles, a rare variant of the mucocele occurring simultaneously with oral lichen planus, are uncommon. This report introduces a case of multiple superficial mucoceles developing with oral lichen planus in a 76-year-old Thai female and provides information to avoid misdiagnosis and over-management of this lesion. Pathogenesis and clinicopathological characteristics of this phenomenon are also discussed.

## 1. Introduction

Superficial mucoceles are an unusual variant of mucocele in the oral cavity that were firstly described by Eveson in 1988 [1]. These lesions are a result of a mucous extravasating phenomenon that particularly originated from minor salivary glands. Superficial mucoceles present as small translucent tense subepithelial vesicles on the oral mucosa and may present as either isolated or multiple lesions. The lesions are frequently located at the labial mucosa, soft palate, retromolar trigone, and buccal mucosa. Unlike usual mucoceles, superficial mucoceles can resolve spontaneously and do not always require treatment except when recurrence is present. Due to the limited number of reports on the characteristics of superficial mucoceles, this disease was often misdiagnosed as other serious oral vesiculobullous diseases such as bullous lichen planus (BLP) and mucous membrane pemphigoid [2].

Previous reports revealed that superficial mucoceles are associated with several events such as trauma, allergy, radiation [3, 4], and various oral diseases such as oral lichen planus (OLP), oral lichenoid lesion from chronic graft-versus-host disease (GVHD), and mycoplasma-induced mucositis [1, 5, 6]. Among these oral diseases, superficial mucoceles are most commonly found with OLP. The latest case series reported by Lv et al. [2] described nine cases of superficial mucoceles developing concomitantly with OLP. All of the patient's superficial mucoceles were refractory to anti-inflammatory treatment [2]. Nowadays, pathogenesis of this phenomenon still remains unknown.

In this report, a 76-year-old female patient presenting with superficial mucoceles and concomitant oral lichen planus was described. The pathogenesis of this phenomenon and treatment are discussed.

## 2. Case Presentation

**Figure 1:**
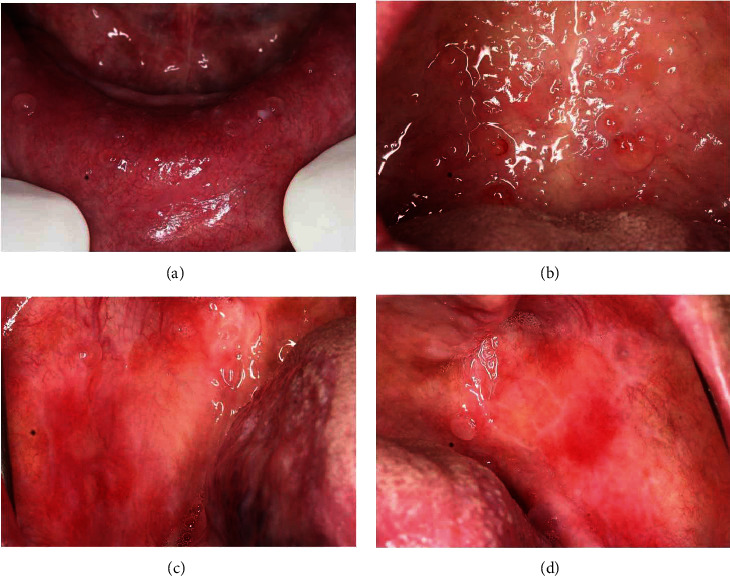
Multiple superficial mucoceles at the lower labial mucosa (a) and at the soft palate with no OLP lesion (b). White striation, erythema, and ulcerated areas at the bilateral buccal mucosa with superficial mucoceles in the adjacent area (c, d).

**Figure 2:**
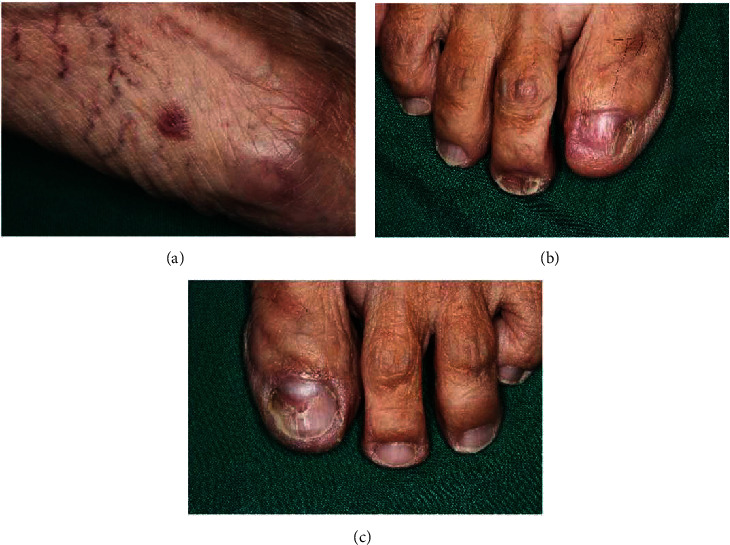
Extraoral examination revealed a polygonal purple papule with white striation on the dorsal surface of the foot (a) and dystrophic toenails (b, c).

A 76-year-old woman was referred by an otorhinolaryngologist to the Faculty of Dentistry, Mahidol University. The patient reported burning sensation in the oral cavity for 6 months, and that was then followed by abundant small vesicles for 3 months. Upon examination, multiple tense and fluid-filled vesicles 1-4 mm in diameter were observed on the upper and lower labial mucosa, buccal mucosa, ventral surface of the tongue, left retromolar trigone, and soft palate (Figures 1(a) and 1(b)). White striation, erythema, and ulcers were found on the edentulous ridge's mucosa and the bilateral buccal mucosa (Figures 1(c) and 1(d)). The laterodorsal area of the tongue showed a depapillated surface. Most of the vesicles were not associated with erythema and white striation. Extraoral examination revealed polygonal and purple papules with white striation on the extensor surface of both legs and the dorsal surface of both feet (Figure 2(a)). Both of her toenails also showed dystrophy (Figures 2(b) and 2(c)). Her past medical history included hypertension, dyslipidemia, and scoliosis. Her medications included losartan, atorvastatin, glucosamine sulfate, calcium carbonate, folic acid, and vitamins B1-6-12.

**Figure 3:**
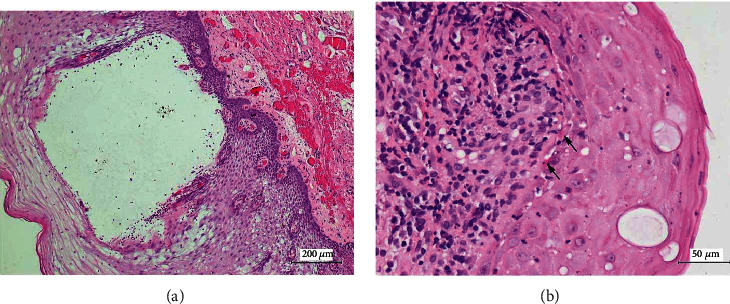
Histopathologic features of superficial mucoceles illustrated an intraepithelial vesicle containing mucin pool with scattered lymphocytes (original magnification, 100x) (a). Histopathologic features of OLP showed basal cell degeneration with subjacent inflammatory cell infiltrate. The arrows indicate civatte bodies (original magnification, 400x) (b).

According to the patient's history and clinical findings, the diagnosis of superficial mucoceles concomitant with OLP was suggested. Incisional biopsies were performed from two sites. The first specimen was taken from the lower labial mucosa, and histopathological examination of superficial mucocele was confirmed. Histopathologically, the specimen showed intraepithelial vesicles containing a mucin pool with scattered lymphocytes (Figure 3(a)). The second specimen representing a red and white lesion was taken from the buccal mucosa. The tissue was bisected and sent for histopathological examination and direct immunofluorescence (DIF). The histological features showed a subepithelial infiltration of lymphocytes and macrophages as well as basal cell degeneration with civatte bodies, confirming the diagnosis of OLP (Figure 3(b)). The DIF finding showed fibrinogen positivity at the basement membrane with a shaggy pattern. Regarding the gold standard of OLP diagnosis [7] and the pathological diagnosis of superficial mucoceles, the definitive diagnosis of superficial mucoceles concomitant with OLP was therefore established.

**Figure 4:**
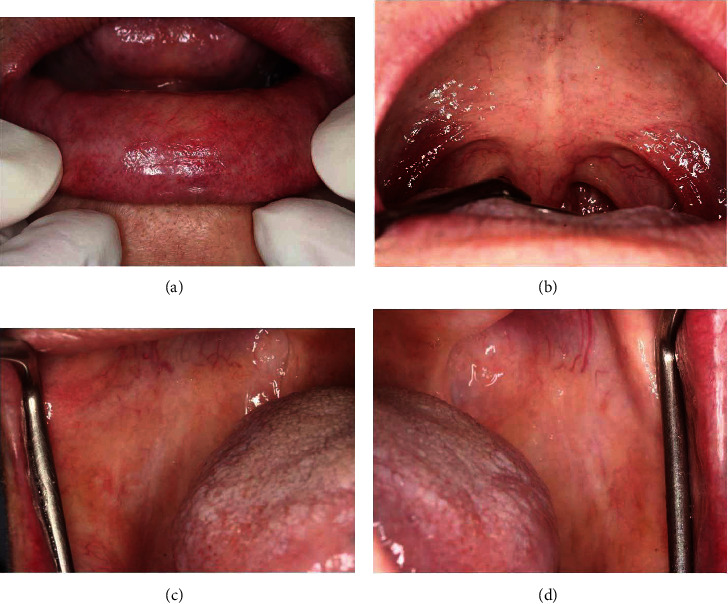
Clinical illustration after 8-month treatment period; complete resolution of superficial mucoceles at the lower labial mucosa (a) and the soft palate (b). Remission of OLP lesions at the bilateral buccal mucosa (c, d).

According to the diagnosis, the high-potency topical steroids including mouth rinse formulated by 0.5 mg dexamethasone dissolved in 10 ml water and 0.1% fluocinolone acetonide in Orabase were prescribed for use three times a day. Additionally, nystatin oral suspension (1 : 100,000 units) was ordered twice a day in conjunction with the topical steroid for prophylaxis of fungal infection. After one month, OLP lesions were remitted, and the superficial mucoceles were decreased in number but remained only at the soft palate and the labial mucosa. The affected areas were temporally relieved as the lesions still developed every meal time, causing patient's discomfort. However, after an 8-month-treatment period, the superficial mucoceles showed complete resolution, and the patient was satisfied with this outcome (Figures 4(a)–4(d)).

## 3. Discussion

Superficial mucoceles arising with OLP are unusual. The vesiculobullous characteristics of superficial mucoceles often lead to a misdiagnosis of BLP. BLP is a rare distinct subtype of OLP caused by epithelial separation. The vesicles of BLP are usually larger, more fragile, and opaque, in comparison to superficial mucoceles [2]. Another remark is that oral BLP is often superimposed with or adjacent to the white striation [8]. In contrast, superficial mucoceles typically develop at the areas of minor salivary glands and can be either within or distant from the OLP lesion [1].

Previous studies reported that multiple superficial mucoceles show a predilection for females and are more frequently found at the age of 30 years and older; that is consistent with our case [1, 9]. However, the most recent case series from Lv et al. did not find any sex predilection [2]. The dermatologic lesion of lichen planus presented in our case resembles that reported by Bermejo et al. [9], whereas other reports did not find any dermatologic condition in the cases of multiple superficial mucoceles occurring with OLP [1, 2, 9]. However, our case showed a difference in location involvement. The superficial mucoceles in our case developed in multiple areas including the labial mucosa, buccal mucosa, ventral surface of the tongue, retromolar trigone, and soft palate. None of the cases from previous reports showed multiple locations [1, 2]. This phenomenon raises a significant disturbance in our patient and confirmed that superficial mucoceles can develop in various oral mucosal sites containing minor salivary glands. In the present case, the soft palate and the ventral surface of the tongue were devoid of OLP lesions. Nevertheless, the most common location of superficial mucocele occurring with OLP reported from previous studies was the soft palate, followed by the buccal mucosa and labial mucosa [2, 9].

It has been proposed that increased intraductal pressure in salivary ducts could lead to ductal obstruction or rupture, resulting in superficial mucoceles [5]. Many authors believed that the salivary ducts of the affected mucosa were blocked or ruptured by lichenoid inflammation, resulting in superficial mucoceles [2, 5, 9]. Some authors proposed that superficial mucoceles occurring with chronic erosive lichen planus were caused by continuous erosion and reepithelialization of the oral mucosa, leading to damage of adjacent salivary ducts of the minor glands [9]. Campana et al. reported a case of recurrent superficial mucoceles developing with cutaneous lichen planus with an absence of OLP [10]. Consistently, the case reported by Lv et al. showed recurrent superficial mucoceles on the soft palate without OLP [2]. They proposed that this occurrence may be related to immunologic disturbance [2]. However, this was unable to explain a cause of superficial mucoceles located far away from the OLP lesion. Lourenco et al. firstly described a term “lichen planus sialadenitis” as a lichenoid infiltration surrounding excretory ducts of affected salivary glands [11]. This report also described that this phenomenon closely resembled lichen planopilaris which is a variant of cutaneous lichen planus affecting hair follicles [11, 12]. In our opinion, the pathogenesis of superficial mucoceles occurring with OLP may be similar to that of lichen planopilaris and is possibly a result of lichen planus sialadenitis. The periductal inflammation of minor salivary glands may be not only related to lichenoid infiltration of the affected mucosal area but also an activation of T-lymphocytes specifically targeting the ductal epithelial antigens, causing ductal inflammation and then superficial mucoceles at distant areas. However, further studies are needed to confirm this notion.

Management of patients suffering from superficial mucoceles with OLP is still a great challenge. The first-line treatment for symptomatic OLP is topical corticosteroid [13]. Previous studies reported that superficial mucoceles were refractory to topical steroids [2, 10] except for betamethasone mouthwash [4]. In our patient, we simulated Motallebejad et al.'s treatment by swishing and spitting out with 0.5 mg dexamethasone dissolved in 10 ml water. The outcome of temporary relief was still a burden for the patient. However, the long-term use of topical steroid conjunction with 0.1% fluocinolone in Orabase and dexamethasone mouth rinse could provide a remission of superficial mucoceles in our case. There are many reports claiming that surgical excision can be used for management of superficial mucoceles [1, 2, 14]. However, the definite treatment for multiple lesions has not been established due to postoperative complications, such as scar and tissue deformation [2]. Jinbu et al. reported that laser vaporization for recurrent multiple superficial mucocele treatment showed no complications [15]. Besides, some authors prefer reassuring patients and periodic observation for asymptomatic condition [2, 10, 16].

In conclusion, superficial mucoceles occurring with OLP are uncommon. We emphasize on recognition of distinct features of this condition to avoid misdiagnosis of other serious oral vesiculobullous diseases. In our case, the long-term use of topical steroids could provide a remission for superficial mucoceles.

## Data Availability

The data presented in this study are available on request from the corresponding author. The data are not publicly available due to ethical issues.
